# Robot-Assisted vs. Open Appendicovesicostomy in Pediatric Urology: A Systematic Review and Single-Center Case Series

**DOI:** 10.3389/fped.2022.908554

**Published:** 2022-05-24

**Authors:** Nikolai Juul, Emma Persad, Oliver Willacy, Jorgen Thorup, Magdalena Fossum, Susanne Reinhardt

**Affiliations:** ^1^Division of Pediatric Surgery, Department of Surgery and Transplantation, Rigshospitalet, Copenhagen University Hospital, Copenhagen, Denmark; ^2^Department of Evidence-Based Medicine and Evaluation, Danube University Krems, Krems, Austria; ^3^Department of Clinical Medicine, University of Copenhagen, Copenhagen, Denmark; ^4^Department of Women’s and Children’s Health, Karolinska Institutet, Stockholm, Sweden

**Keywords:** pediatrics, urology, urinary diversion, cystostomy, robotic surgical procedures

## Abstract

**Introduction:**

Appendicovesicostomy (APV) is the preferred choice of continent catheterizable channels in pediatric urology. The introduction of robot-assisted laparoscopic techniques has been correlated to superior cosmesis and convalescence and is now increasingly implemented for APV procedures. We aimed to perform a systematic review of the literature comparing open vs. robotic APV regarding possible differences in postoperative outcomes and to evaluate these findings with our own initial experiences with robotic APV compared to our previous open procedures.

**Methods:**

We evaluated the first five patients undergoing robotic APV at our institution and compared 1-year outcomes with a consecutive series of 12 patients undergoing open APV. In a systematic literature review, we screened studies from PubMed, EMBASE, and CENTRAL comparing open and robotic APV in pediatric urology (current to December 2021) and performed meta-analyses on postoperative outcomes comparing the two groups and evaluated the grade of evidence.

**Results:**

We found significantly shortened postoperative length of stay in the robotic group (*p* = 0.001) and comparable 1-year complication rates in robotic vs. open APV patients. We systematically screened 3,204 studies and ultimately included three non-randomized studies comparing postoperative outcomes of robotic and open APV for quantitative analysis. The open and robotic approaches performed equally well regarding overall postoperative complications, surgical reintervention, and stomal stenosis. Two of the included studies reported comparable stomal continence rates and shortened postoperative length of stay in the robotic group, in agreement with the findings in our own series.

**Conclusion:**

Robotic APV is equally safe to the conventional open approach with additional advantages in postoperative hospitalization length.

## Introduction

The trans-appendicular continent cystostomy, also referred to as the appendicovesicostomy (APV), was first described by Mitrofanoff (1). Although numerous variations and modifications of the procedure have since been proposed, the procedure maintains its popularity due to its basic principles: the appendix vermiformis is detached from the cecum, whilst preserving the mesenteric blood supply, and used as a conduit connecting the urinary bladder to a skin stoma. The procedure was originally designed for children with neurogenic bladder dysfunction, as an alternative route for catheterization for those who were unable or unwilling to utilize the urethra, or in cases where bladder neck closure was necessary to achieve continence.

Since then, the indications for the procedure have expanded to various selected cases, including urethral valves, prune belly and bladder exstrophy, performed either as an isolated procedure enabling patients to perform clean intermittent catheterization (CIC) or in relation with concomitant bladder augmentation.

Since the turn of the century, an increasing number of publications have advocated for the excellent results achieved by performing the procedure laparoscopically compared to the original open approach (2–10). The benefits of the laparoscopic approach in most surgical procedures, including the APV, include decreased postoperative pain, shorter hospital stay, and improved cosmesis (10).

In agreement with other reports in pediatric and adult urology, we have previously found that robot-assisted laparoscopic surgery may provide additional benefits compared to the laparoscopic approach, especially when the procedure includes a substantial amount of suturing (11–14). The improved surgical handling when performing robotic procedures involving suturing may explain the shorter operating time compared to similar laparoscopic operations (15–18). With this knowledge, the rationale to move directly from open surgery to robotics is reasonable, if the technique is accessible.

Although isolated APV is only indicated a few times annually in most pediatric urology institutions, general access to robotic services in university centers is becoming increasingly common, warranting an analysis of potential benefits, and harms.

This study aimed to evaluate whether transitioning from open APV directly to the robot-assisted procedure was feasible. We hypothesized that the proposed advantages of robotic surgery compared to open surgery would lead to successful procedures and improved patient outcomes, in line with what has been reported for the laparoscopic approach. We, therefore, performed an institutional case series analysis and placed our findings within the context of the literature by performing a systematic review and quantitative analyses of studies comparing robotic and open APV.

## Materials and Methods

### Case Series

Robotic APV was introduced at our institution in 2015, and in this study, we chose to report on the first five patients undergoing the isolated APV procedure. All procedures were performed by the same surgeon, using the same approach each time: A transperitoneal three-port setup, with a fourth port placed in the lower right quadrant for final stoma placement. The appendix was anastomosed to the posterior bladder wall and covered by bladder muscle to create a submucosal tunnel for continence, and an indwelling catheter was left in the APV for 3 weeks postoperatively before the CIC regimen was established.

In the open access group, we included the last 12 consecutive patients undergoing open isolated APV between 2007 and 2014, before the introduction of the robotic technique. In this group, three different surgeons were involved individually. Intraperitoneal access was gained *via* a Pfannenstiel incision and the stoma was placed in the lower right quadrant. In accordance with the robotic procedure, the appendix was anastomosed to the posterior bladder wall, with the bladder muscle wall for continence, and an indwelling catheter left in the conduit at least 2 weeks postoperatively before the start of CIC.

For both procedures, the operating time from skin incision to final skin closure was recorded perioperatively and stored prospectively in the hospital patient charts.

For comparison between the two groups, follow-up time up to 1 year after intervention was accessed from the patient records and included: operating time, postoperative length of stay, and 1-year postoperative complications according to the Clavien-Dindo classification (19). Statistical differences in baseline demographics, adverse events and primary outcomes were assessed with Fisher’s exact test and student’s *t*-test for binary and continuous variables, respectively, and *p* < 0.05 was considered statistically significant.

### Systematic Review

A systematic review was performed to find comparative data on surgical operative times, postoperative complications, and length of stay from robotic and open APV procedures. Inclusion and exclusion criteria were registered in the PROSPERO database for systematic review protocols (ID CRD42021289515) and can be found in Supplementary Table 1 (20). We searched PubMed, CENTRAL, and EMBASE current to December 7, 2021 using a search strategy that included a combination of free text and controlled vocabulary (e.g., MeSH) (Supplementary Table 2). The search period and search languages were not limited. We additionally searched the reference lists of similar systematic reviews and other studies, tagged as background articles, for crosschecking with our electronic searches. All titles and abstracts were dually and independently reviewed by two team members for eligibility against our inclusion/exclusion criteria in the Covidence systematic review software (Veritas Health Innovation, Melbourne, Australia). All eligible studies were then evaluated at the full-text level independently.

To assess the risk of bias in non-randomized studies, we used ROBINS-I (Risk Of Bias In Non-randomized Studies of Interventions) (21). We graded the strength of evidence-based on the guidance established by the GRADE (Grading of Recommendations Assessment, Development, and Evaluation) Working Group (22). For quantitative analyses (i.e., meta-analysis) we used random-effects models to estimate pooled or comparative effects in odds ratios (OR) with corresponding confidence intervals (CI) using RevMan Web (23).

## Results

### Case Series

The gender distribution was comparable in the two groups, with the majority being male (80 vs. 67% in robotic and open, respectively). Patients in the robotic group were older (12.08 vs. 5.75 years, *p* = 0.02). The most common indication for APV in both groups was neurogenic bladder, more often due to spina bifida (i.e., myelomeningocele). Operating time for robotic APV varied between 208 and 301 min (mean 249 ± 35) and did not differ significantly from the open procedure (mean 231 ± 105 min, *p* = 0.719). No perioperative complications were recorded in either groups, however, one patient in the open APV group later required surgical reintervention under general anesthesia due to a deep wound infection, which did not occur in the robotic group. Evaluating the difference in postoperative length of hospitalization revealed a significantly shorter length of stay in the robotic group (2.6 vs. 9.3 days, *p* = 0.001). During the 1-year follow-up period, we found comparable rates of overall postoperative complications in the two groups (40% in the robotic group vs. 33% in the open group, *p* = 0.858), with the majority of complications not requiring surgical reintervention (Clavien-Dindo grade IIIb or lower). Even though we found a higher proportion of channel stenosis in the robotics group (40% in the robotic group vs. 25% in the open group), this difference was not statistically significant (*p* = 0.601). Stomal continency was the same in both groups (80% for robotic vs. 83% for open, *p* = 0.891). Baseline demographics and 1-year postoperative outcomes for both patient groups are summarized in Table 1.

**TABLE 1:** Baseline characteristics and 1-year postoperative outcomes after isolated appendicovesicotstomy in our institution.

	Robotic	Open	*p*-value
No. of patients	5	12	
Male gender (%)	4 (80)	8 (67)	1.000
Mean age at surgery (years)	12.08 (± 5.03)	5.75 (± 4.45)	0.020
**Primary diagnosis**			
Myelodysplasia	3	4	
Posterior urethral valve	1	2	
Bladder exstrophy		2	
Prune belly		1	
Imperforated anus		1	
Trauma	1	1	
Other		2	
Overall complications (%)	2 (40)	4 (33)	1.000
**Clavien-Dindo grade**			
I			
II	1 (catheterization problems)	2 (catheterization problems, recurrent UTI)	
IIIb	1 (stomal granuloma)	1 (stomal granuloma)	
IIIa		1 (deep wound infection)	
IV			
V			
Operative time (minutes)	249 (± 35)	231 (± 105)	0.719
Postoperative length of stay (days)	2.6 (± 0.89)	9.3 (± 3.75)	0.001
Stomal incontinence (%)	1 (20)	2 (17)	1.000
Channel stenosis (%)	2 (40)	3 (25)	0.601

*Robot-assisted and open groups are presented, respectively. Absolute numbers are presented with percentage (%), means are presented with standard deviation (SD).*

### Systematic Review

**FIGURE 1:**
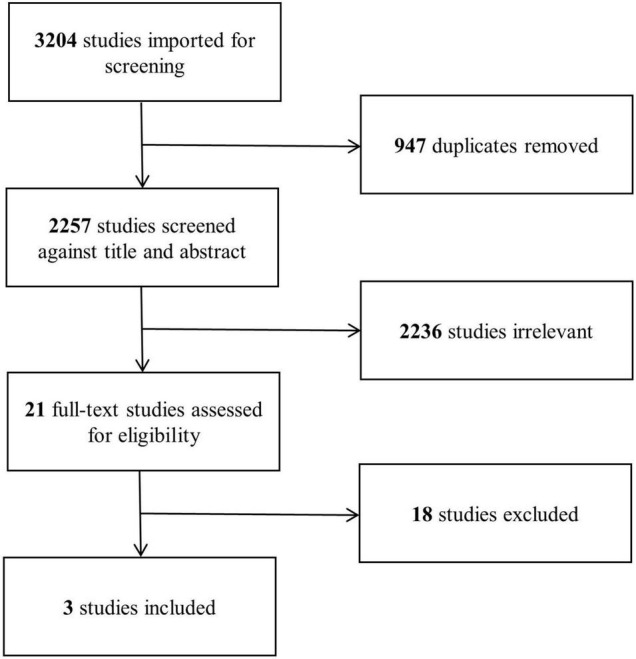
PRISMA flow diagram of systematic review study selection.

Our search strategy yielded a total of 2,257 studies after removal of duplicates, of which 21 were found eligible for full-text assessment (Figure 1). Eighteen studies reporting surgical outcomes of APV were excluded for not including an open procedure comparator group. Three studies (24–26), each comparing peri- and postoperative outcomes of robotic and open APV from within the same institution, were ultimately included for analysis (Table 2). This resulted in a total study population of 156 patients (83 robotic and 73 open) with a mean age ranging from 6.7 to 11.9 years and a median follow-up time from 1.2 to 4.4 years. All of the included studies were North American single-center studies, presenting non-randomized results from initial robotic APV experiences compared to retrospectively assessed open APV, similar to the results of our own series. Each of the studies provided detailed surgical results, and the general risk of bias related to adverse outcomes was considered low (Supplementary Table 3). When evaluating the evidence level according to GRADE, we found no serious inconsistency, no serious indirectness or impression of publication bias, however, the overall level of evidence gathered from the sum of these studies was graded as low, mainly due to the non-randomized study design in all included studies (Supplementary Table 4).

**TABLE 2:** Final studies included in systematic review.

	Nguyen et al. (24)		Grimsby et al. (25)		Galansky et al. (26)	
	Robotic	Open	Robotic	Open	Robotic	Open
No. of patients	10	10	39	28	34	35
Mean age (years)	11.9	10.6	9.9	6.7	10.8	7.3
Median follow-up (years)	1.2	1.6	3.4	2	4.4	NR
Concomitant procedures	0	NR	18	19	22	32
Postoperative hospitalization (days)	5	8	NR	NR	6.8	13
Surgical reinterventions	1	NR	13	8	8	7
Postoperative complications	2	4	10	8	13	15
Stomal stenosis	0	2	1	4	7	8
Stomal incontinency	1	NR	4	4	3	1

*NR, not reported.*

**FIGURE 2:**
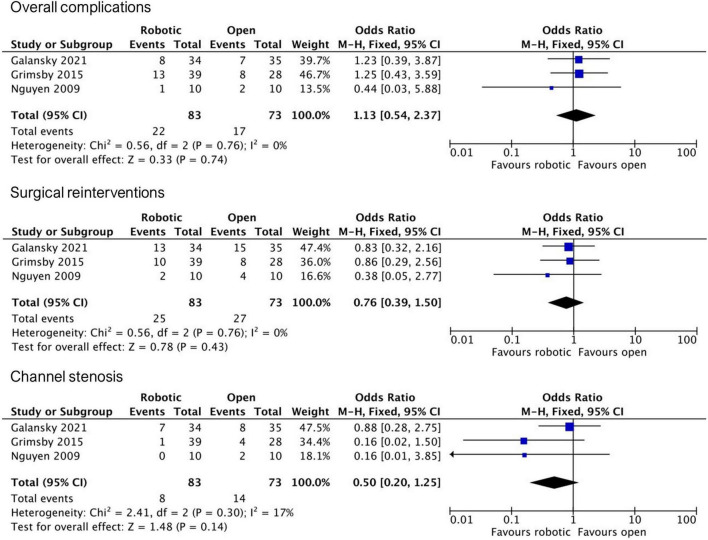
Meta-analyses of postoperative outcomes of robotic vs. open APV.

Based on the final study material, we conducted three independent meta-analyses comparing robotic and open APV outcomes regarding (1) overall complications, (2) surgical reinterventions, and (3) stomal stenosis (Figure 2). All three included studies reported similar complication rates between the two groups, with a pooled OR of 1.13 (95% CI, 0.54–2.37). Regarding surgical reinterventions, all studies reported lesser rates in the robotic group, however, with wide confidence intervals overlapping non-significance, resulting in a pooled OR of 0.76 (95% CI, 0.39–1.50). Favorable tendencies for the robotic group in all studies were also reported concerning postoperative stomal stenosis, however not found to be statistically significant, with a pooled OR of 0.5 (95% CI, 0.20–1.25).

As only two of the studies reported on postoperative length of stay, this endpoint was not used for meta-analysis. Yet, both the studies reported shorter length of stay in the robotic group in alignment with our own results (6.8 vs. 13 days in Galansky et al. and 5 vs. 8 days in Nguyen et al.). Another surgical outcome, stomal continency, was also only reported in two of the studies, both reporting comparable rates between the two groups (90% for robotic vs. 86% for open in Grimsby et al. and 91% for robotic vs. 97% for open in Galansky et al.).

## Discussion

We performed a consecutive analysis of our outcomes after introduction of the robotic APV technique and related our results to an international context by performing a systematic review of the publications on the topic. In our case series, robotic APV patients demonstrated favorable 1-year postoperative outcomes in terms of complication rates and postoperative length of stay. Our operating time was fully in accordance with that reported in the literature (27). Further, when compared to laparoscopic procedures, our operating time and hospitalization length proved satisfactory according to previously published series of isolated laparoscopic APV, that report median operating times ranging from 139 to 255 min and postoperative length of hospitalization ranging from 3.5 to 7 days (3–5, 10, 28–30). Furthermore, we systematically reviewed prior studies directly comparing robotic and open APV, and quantitatively analyzed differences in adverse outcomes. A general tendency revealed equal complication rates between robotic and open APV and a shorter postoperative length of stay. While this, to our knowledge, is the first systematic review and meta-analysis performed on this patient group, our review outcome was limited to only three non-randomized single-center studies and the overall level of evidence concerning adverse outcomes of robotic vs. open APV was considered low. Nevertheless, as in many other fields of surgical advances, the nature of these procedures might never allow for fully controlled randomized trials (31).

Our case series has limitations, most notably the small size of our robotic series and the risk of uncontrolled confounders, due to the retrospective setting. While we did not find a statistically significant difference in many of our postoperative outcomes, the rates of stomal stenosis present disparity between the groups and statistical insignificance could be due to underpowered data. By only comparing patients undergoing isolated APV, we have minimized this risk, as the addition of concomitant augmentations is known to increase the length of surgery and risk of postoperative complications (32). Although the two patient groups in our case series differ in age, previous studies have demonstrated that increasing age does not impact APV complication rates independently (33, 34), one reason may be that the average appendix reaches adult proportions early in life (35). The 1-year follow-up in our series cannot account for all potential complications in these patients, as complications can occur life-long (36), still, most APV related surgical complications will occur within this period (37, 38).

Since robot-assisted laparoscopic APV was first reported in 2004 in a young boy presenting with a condition of congenital posterior urethral valves (39), more reports rapidly confirmed its feasibility, with or without concomitant augmentation ileocystoplasty, and for conduits for antegrade enemas in different conditions (spina bifida, prune belly syndrome, multiple sclerosis) (27, 40–46). Overall, the reported stomal continence is considered successful with successful clean intermittent catheterizations. Some series also report minor additional procedures to revise the APV channel or inject hyaluronic acid in cases of persisting leakage, which is comparable to the results observed in the open surgery population (47–52).

The average cost per procedure in adult abdominal surgery related to the DaVinci system alone has been estimated to be approximately 3,568 USD (reported 2018), including implementation and maintenance but not any other procedure-related costs (53). In comparison, the total costs of the corresponding open procedures have been estimated to 3-7000 USD all expenses included (with instruments accounting for less than 20%) (54). This illustrates that high perioperative costs can be counter-balanced by shorter hospital stay and sequentially shorter parental- and child leave from work and school, respectively.

Apart from pyeloplasty, most pediatric urology robotic procedures, including isolated robotic APV without bladder augmentation, are not often performed and therefore considered rare cases in most institutions. Even in a large multicenter study of 73 cases without augmentation from five institutions, data indicates that equal distribution from 2008 onward would only result in few (1–2) isolated robotic APVs annually at each institution (27).

While many pediatric urology institutions do not have access to robotic systems, the laparoscopic procedure is still considered a safe alternative with similar benefits in postoperative pain, cosmesis, and hospitalization. However, when a robotic surgery program is available and used by a dedicated pediatric urologist, the shallow learning curve adjusting from open to robot-assisted procedures becomes advantageous for both hospital and patient-relevant outcomes.

## Conclusion

Our case series and systematic review of the literature indicate slightly shorter postoperative length of hospital stay following robotic surgery. However, the methods share similar outcomes. When a robotic surgery program is available, it is safe, and justifiable to use the robotic approach.

## Data Availability Statement

The raw data supporting the conclusions of this article will be made available by the authors, without undue reservation.

## Ethics Statement

The studies involving human participants were reviewed and approved by the legal department of our institution (Rigshospitalet, Copenhagen, Denmark. File no. 22008114). Written informed consent from the participants’ legal guardian/next of kin was not required to participate in this study in accordance with the national legislation and the institutional requirements.

## Author Contributions

NJ, EP, OW, JT, MF, and SR developed the study design and drafted the final manuscript. JT and SR obtained clinical data for the case series. NJ and EP conducted the systematic review and performed the meta-analysis. All authors contributed to the article and approved the submitted version.

## Conflict of Interest

The authors declare that the research was conducted in the absence of any commercial or financial relationships that could be construed as a potential conflict of interest.

## Publisher’s Note

All claims expressed in this article are solely those of the authors and do not necessarily represent those of their affiliated organizations, or those of the publisher, the editors and the reviewers. Any product that may be evaluated in this article, or claim that may be made by its manufacturer, is not guaranteed or endorsed by the publisher.
