# Supplementary material for: Robot-Assisted vs. Open Appendicovesicostomy in Pediatric Urology: A Systematic Review and Single-Center Case Series
Source: Front Pediatr. 2022 May 24;10:908554. doi: 10.3389/fped.2022.908554 (PMC9171498; doi:10.3389/fped.2022.908554)
Supplement: Supplementary file 1 [file Data_Sheet_1.docx]

**Supplementary material**

Table S1. Inclusion and exclusion criteria. APV: appendicovesicostomy; RCTs: randomized-controlled trials

| Category |  | |
| --- | --- | --- |
|  | Inclusion | Exclusion |
| Population | Children and adolescents having performed APV |  |
| Intervention | Robot-assisted laparoscopic APV |  |
| Control | Open APV |  |
| Outcomes | Duration of procedure  Length of hospitalization  Incontinency rate  Stomal stenosis  Surgical channel revision  Quality of life assessments | Studies that do not include at least one of the outcomes listed under the inclusion criteria |
| Setting |  |  |
| Timing of intervention | No limitations |  |
| Geography | No limitations |  |
| Language | No limitations |  |
| Study design | RCTs  Systematic review and meta-analyses (for crosschecking)  Nonrandomized controlled trials  Prospective cohort studies  Retrospective controlled cohort studies  Case-control studies  Case series | Case reports  Studies without a control group |

Table S2. Search terms

| **PubMed Query – December 7, 2021** | **Results** |
| --- | --- |
| ("Adolescent"[Mesh] OR adolescent*[tiab] OR infant*[tiab] OR newborn OR neonate*[tiab] OR child*[tiab] OR teen*[tiab] OR pediatr*[tiab] OR paediatr*[tiab]) AND (((((mitrofanoff) OR ("monti channel")) OR (appendicovesicostomy)) OR (continent catheterizable channel)) OR (((("Cystostomy"[Mesh]) OR (Cystostomy)) OR (Cystostomies)) OR (Vesicostomy OR Vesicostomies))) |  |
|  | 2786 |
| **Embase Query – December 7, 2021** | **Results** |
| ('Adolescent'/exp OR Adolescent*:ti,ab OR infant*:ti,ab OR newborn*:ti,ab OR neonate*:ti,ab OR child*:ti,ab OR teen*:ti,ab OR pediatr*:ti,ab OR paediatr*) AND (mitrofanoff:ab,ti,kw OR 'monti channel':ab,ti,kw OR appendicovesicostomy:ab,ti,kw OR 'continent catheterizable channel':ab,ti,kw OR Cystostomy:ab,ti,kw OR Cystostomies:ab,ti,kw OR Vesicostomy:ab,ti,kw OR Vesicostomies:ab,ti,kw) | 2897 |
| **Central Query – December 7, 2021** | **Results** |
| Adolescent* OR infant* OR newborn OR neonate* OR child OR children OR teen* OR pediatric* OR paediatric* AND mitrofanoff OR monti channel OR appendicovesicostomy OR continent catheterizable channel OR Cystostomy OR Cystostomies OR Vesicostomy OR Vesicostomies | 3204 |

Table S3. Risk of bias in adverse outcomes

| **Risk of bias** | | | | | | | | |
| --- | --- | --- | --- | --- | --- | --- | --- | --- |
| **Author, year** | **Bias due to confound-ding** | **Bias in selection of participants into the study** | **Bias in classification of interventions** | **Bias due to deviations from intended interventions** | **Bias due to missing data** | **Bias in measurement of outcomes** | **Bias in selection of the reported result** | **Overall risk of bias** |
| Nguyen, 2009 | Low | Moderate | Low | Moderate | Low | Low | Low | Low |
| Grimsby, 2015 | Low | Moderate | Low | Moderate | Low | Low | Low | Low |
| Galansky, 2021 | Low | Moderate | Low | Moderate | Low | Low | Low | Low |

Table S4. Evidence level according to GRADE. A) Inconsistency refers to an unexplained heterogeneity of results. B) Indirectness refers to unexplained deviation of the population, intervention and outcome measures from the research question. C) Imprecision refers to when the confidence interval includes both benefit and harm.

| **GRADE evidence profile** | | | | | | | | | | |
| --- | --- | --- | --- | --- | --- | --- | --- | --- | --- | --- |
| **Outcome** | **Number of studies** | **Number of participants** | **Risk of bias** | **Inconsistency^a^** | **Indirectness^b^** | **Imprecision^c^** | **Publication bias** | **Summary effect size/outcome** | **Certainty of the evidence** |  |
| Postoperative complications | 3 | 156 | Low | No serious inconsistency | No serious indirectness | No serious imprecision | Not detected | See Table 2 | Low |  |
| Surgical reinterventions | 3 | 156 | Low | No serious inconsistency | No serious indirectness | No serious imprecision | Not detected | See Table 2 | Low |  |
| Stomal stenosis | 3 | 156 | Low | No serious inconsistency | No serious indirectness | No serious imprecision | Not detected | See Table 2 | Low |  |
